# Evidence of carbamate resistance in urban populations of *Anopheles gambiae s.s.* mosquitoes resistant to DDT and deltamethrin insecticides in Lagos, South-Western Nigeria

**DOI:** 10.1186/1756-3305-5-116

**Published:** 2012-06-11

**Authors:** Adedayo O Oduola, Emmanuel T Idowu, Muyiwa K Oyebola, Adedapo O Adeogun, Judith B Olojede, Olubunmi A Otubanjo, Taiwo S Awolola

**Affiliations:** 1 Molecular Entomology and Vector Control Research Laboratory, Public Health Division, Nigerian Institute of Medical Research, Akoka, Lagos, Nigeria; 2 Department of Zoology, University of Lagos, Akoka, Lagos, Nigeria

**Keywords:** Carbamate, DDT, Pyrethroids, Insecticide resistance, Urban, *Anopheles gambiae* mosquitoes, Lagos, Nigeria

## Abstract

**Background:**

Resistance monitoring is essential in ensuring the success of insecticide based vector control programmes. This study was carried out to assess the susceptibility status of urban populations of *Anopheles gambiae* to carbamate insecticide being considered for vector control in mosquito populations previously reported to be resistant to DDT and permethrin.

**Methods:**

Two – three day old adult female *Anopheles* mosquitoes reared from larval collections in 11 study sites from Local Government Areas of Lagos were exposed to test papers impregnated with DDT 4%, deltamethrin 0.05% and propoxur 0.1% insecticides. Additional tests were carried out to determine the susceptibility status of the *Anopheles gambiae* population to bendiocarb insecticide. Members of the *A. gambiae complex*, the molecular forms, were identified by PCR assays. The involvement of metabolic enzymes in carbamate resistance was assessed using Piperonyl butoxide (PBO) synergist assays. The presence of kdr-*w/e* and ace-1R point mutations responsible for DDT-pyrethroid and carbamate resistance mechanisms was also investigated by PCR.

**Results:**

Propoxur resistance was found in 10 out of the 11 study sites. Resistance to three classes of insecticides was observed in five urban localities. Mortality rates in mosquitoes exposed to deltamethrin and propoxur did not show any significant difference (P > 0.05) but was significantly higher (P < 0.05) in populations exposed to DDT. All mosquitoes tested were identified as *A. gambiae s.s* (M form). The *kdr -w* point mutation at allelic frequencies between 45%-77% was identified as one of the resistant mechanisms responsible for DDT and pyrethroid resistance. *Ace-1R* point mutation was absent in the carbamate resistant population. However, the possible involvement of metabolic resistance was confirmed by synergistic assays conducted.

**Conclusion:**

Evidence of carbamate resistance in *A. gambiae* populations already harbouring resistance to DDT and permethrin is a clear indication that calls for the implementation of insecticide resistance management strategies to combat the multiple resistance identified.

## Background

Malaria is a major public health problem and *Anopheles gambiae* is one of the major vectors of this disease in sub-Saharan Africa [1]. In most urban communities of Lagos State, *A. gambiae* mosquitoes are found breeding abundantly in widely available sun-lit stagnant pools of water created by flooding resulting from numerous blocked drains during the rainy season [2]. The abundance of these vector species during the rainy season between April and October usually coincides with the peak of the malaria transmission period. The current best practices in vector control include the use of Long Lasting Insecticide Nets (LLIN), Indoor Residual Spraying (IRS) and Larviciding [3].

In recent times, IRS is being adopted and scaled up to protect the entire household and community members who possibly have no access to treated bed nets. It is also being scaled up to address the problem of slow improvement in the utilization of bed nets in Africa [4,5].

Out of the four classes of insecticides (organochlorine, pyrethroids, carbamates and organophosphates) approved for malaria vector control [6] only the pyrethroid insecticides are recommended for the treatment of bed nets, the others are applied for IRS. In Nigeria, pyrethroids are currently the most widely used insecticides either in the treatment of bed nets or for IRS. Its usage as an insecticide of choice for malaria vector control purposes relies on their known efficacy at low dosage, relative safety for humans and non-target organisms, excito-repellent properties, rapid rate of knock-down and residual killing effects [7]. The lower cost of procurement and application when compared to carbamate is another reason for its choice during vector control programmes [8]. Though the World Health Organisation (WHO) has approved the use of dichlorodiphenyltrichloroethane (DDT) for public health [9], the non usage of DDT by some countries may not be farfetched from issues on human and environmental safety [10]. Insecticide mosaic and rotational use of different classes has been proposed for resistance management [11]. With the widespread resistance to pyrethroid and organochlorine, carbamate is one of the possible alternatives that can be considered to combat pyrethroid-DDT resistance, mainly because of its separate mode of action. Pyrethroids and DDT insecticides share the same target site; hence resistance to one class may possibly indicate resistance to another. In terms of cost of application on a house to house basis, DDT still remains the least expensive of all the three classes of insecticides [8].

Unfortunately, resistance of *Anopheles* species to several classes of insecticide; DDT, carbamates, organophosphates and pyrethroids has been reported in several African countries [12]. In Nigeria, resistance of *Anopheles gambiae*, the major malaria vector to organochlorine and pyrethroid insecticides is well documented [13-15]. However, there is paucity of information on the insecticide resistance status of the field strain of *A. gambiae* in Nigeria to carbamate insecticide.

The objective of this study is to provide information on the susceptibility status of *Anopheles gambiae* to carbamate that has not been used in vector control and also to investigate the possibility of co-existence of multiple insecticide resistance in the same population of *A. gambiae* that was previously reported to be resistant to permethrin and DDT [15]*.* It is hoped that findings from this study will promote and improve effective vector control decision making.

## Methods

### Study area

**Figure 1:**
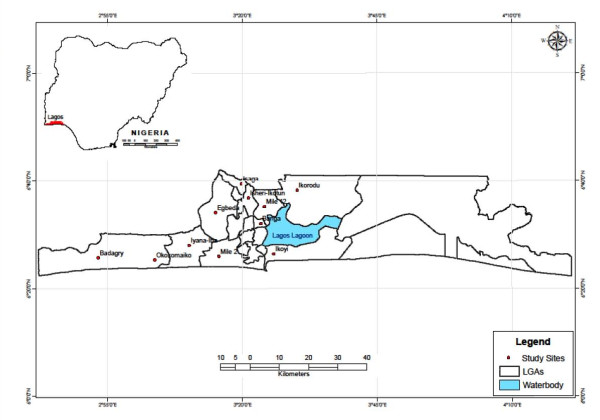
Map of Lagos showing the study localities, displayed in the inset is the map of Nigeria.

The study was carried out in 11 communities from 10 Local Government Areas (LGAs) in Lagos State (3° 24’ E and latitude 6° 27’ N). Lagos is a city in south-western Nigeria (Figure 1). The study communities and corresponding LGAs are shown in Figure 1. The Lagos metropolitan area spreads over much of Lagos State (3500 km^2^), and occupies Lagos Island, Ikoyi Island, and Victoria Island, as well as a large area on the mainland, all connected by a series of bridges and freeways. Lagos is Nigeria’s largest city, chief port, and principal industrial, economic and cultural center. Lagos has a very diverse and fast-growing population, resulting from heavy and ongoing migration to the city from all parts of Nigeria as well as neighbouring countries for trading and employment opportunities.

**Table 1:** **Knockdown times (KDTs) for*Anopheles gambiae s.l.*in urban Lagos after exposure to different insecticides**

		**DDT (4%)**			**Deltamethrin(0.05%)**			**Propoxur (0.1%)**		
**LGA**	**Study sites**	**n**	**KDT**_**50**_**(95%CI)**	**KDT**_**95**_**(95%CI)**	**n**	**KDT**_**50**_**(95%CI)**	**KDT**_**95**_**(95%CI)**	**n**	**KDT**_**50**_**(95%CI)**	**KDT**_**95**_**(95%CI)**
**Badagry**	Badagry	82	a	a	80	31.31 (22.65-45.58)	96.21 (59.56-411.52)	60	a	a
**Amuwo Odofin**	Mile 2	60	a	a	60	46.03 (39.23-57.71)	124.36 (87.60-254.63)	60	a	a
**Ifako Ijaiye**	Isaga	63	a	a	60	53.20 (47.21-62.46)	171.11 (126.43-275.01)	60	a	a
**Somolu**	Bariga		b	b	88	39.49 (36.30-42.91)	59.82 (54.65-67.73)	87	59.90 (56.32-64.76)	91.88 (83.41-104.00)
**Ikorodu**	Ikorodu	80	12. (11.25-14.88)	19.78 (48.96-78.73)	93	49.54 (47.47-51.87)	73.84 (69.48-79.60)	83	42.52 (40.72-44.38)	62.21 (59.08-66.18)
**Eti Osa**	Ikoyi	80	b	b	85	48.13 (45.64-51.10)	88.32 (78.21-105.15)	84	42.97 (40.03-46.01)	60.80 (56.25-67.78)
**Kosofe**	Mile 12	80	b	b	100	50.99 (39.13-42.92)	67.96 (62.62-75.87)	80	38.93 (37.17-40.75)	58.84 (55.82-62.63)
**Alimoso**	Egbeda	111	a	a	90	38.40(35.99-41.10)	91.69 (80.17-109.19)	88	a	a
**Ojoo**	Iyana-Iba	86	a	a	97	50.47 (47.44-54.30)	106.63 (91.69-132.05)	89	38.40 (36.35-40.58)	75.66 (68.32-86.40)
**Ojoo**	Okokomaiko	80	c	c	87	41.32 (38.60-44.51)	101.62 (87.25-124.64)	94	46.03 (43.53-48.96)	92.55 (81.57-110.02)
**Alimoso**	Isheri-Ikotun	80	c	c	93	a	244.45 (170.49-438.37)	88	55.18 (46.38-97.48)	89.97 (66.69-83.99)
**Total**		**802**			**933**			**873**		

n = numbers of mosquitoes exposed a = 50% knockdown was not obtained within the 60 minutes of exposure period b = No knockdown effect.

c = Lower and upper confidence limits could not be estimated due to large KdT value.

### Mosquito larval collection and rearing

*Anopheles gambiae s.l.* larvae were collected in 11 study communities namely: Badagry, Mile 2, Isaga, Bariga, Ikorodu, Ikoyi, Mile 12, Egbeda, Iyana Iba, Okokomaiko, Isheri-Ikotun selected randomly, from June to August 2010. At each locality chosen, *Anopheline* larvae were collected from various natural breeding sites including ground pools, gutters, puddles and abandoned potholes. Water was scooped using a plastic scoop and poured into small transparent plastic bowls. A strainer was used to sieve and pool together the third and fourth instar larvae in order to have sufficient adult emergence of the same physiological age. The bowls were scrutinized for presence of unwanted organisms or predators; if any were found, a pipette was used to remove them. The Global Positioning System (GPS) was used to establish the coordinates of the study sites. The mosquito larvae collected were transported in well labelled plastic bottles to The Molecular Entomology and Vector Control Unit Insectary at the Nigerian Institute of Medical Research, Yaba Lagos where they were maintained at 28 ± 2 C and 72 ± 5% relative humidity.

### Mosquito identification

The mosquitoes exposed in the tests were examined under the dissecting microscope (Olympus SZ 40) and identified to species level using morphological identification keys [16].

### Insecticide susceptibility tests

Insecticide susceptibility tests were performed using the WHO standard procedures and test kits for adult mosquitoes [17]. Test papers impregnated with recommended diagnostic concentrations of 4% DDT, 0.05% deltamethrin, 0.1% propoxur and 0.1% bendiocarb were used. The Kisumu reference strain susceptible to all insecticide classes was used to establish the potency of the treated papers before the exposures were carried out using wild samples. Tests were carried out using 2–3 day old, non-blood fed female mosquitoes. For each insecticide, 3–4 replicates of 20 to 25 females were exposed to the insecticide-impregnated test papers in the test tubes for 1 hour. Considering the homogeneous identity of populations of *A. gambiae* observed in Lagos, additional tests were carried out to determine the effect of PBO synergist combinations with bendiocarb and propoxur on the mortality of *A. gambiae* collected. Adult female mosquitoes were pre-exposed to 4% PBO paper for 1hour following which they were immediately exposed to bendiocarb and propoxur for another 1hour. Final mortality was recorded after 24 hours and the results compared with those without PBO. Sub-populations were also exposed to DDT and deltamethrin to confirm the susceptibility status of the population to both insecticides as earlier reported. The number of mosquitoes that were knockdown was recorded every ten minutes during the 1-hour exposure period. Mosquitoes were then transferred into holding tubes and supplied with a 10% sugar solution. Mortality was recorded 24 hours after exposure. Controls were also set up by exposing the field strain to untreated papers. The tests results were discarded if mortality in the control group was over 20%. If it was between 5 and 20%, mortality rates were corrected using Abbott's formula [18]. Resistant and susceptible mosquitoes were preserved separately in eppendorf tubes filled with desiccated silica gel.

### DNA extraction, molecular identification, *kdr-w/e* and ace-1^R^ mutations of acetyl cholinesterase gene

Genomic DNA was extracted from sub samples of mosquitoes that were randomly selected from a pool of susceptible and resistant populations from each study location [19]. Extracted DNA was subjected to Polymerase Chain Reaction – Restriction Fragment Length Polymorphism (PCR- RFLP) assay to simultaneously identify members of *Anopheles gambiae* complex and the molecular forms [20]. The *kdr-w/e* and insensitive G119S (Ace*. 1*^*R*^) point mutation recognition assays were carried out on at least 20 sub - samples selected from each population [21-23].

### Interpretation of data and statistical analysis

Percentage knockdown to the three insecticides and percentage mortality were determined for the mosquitoes from each of the study localities. The WHO [17] criteria was used to evaluate the resistance/susceptibility status of the mosquitoes. By the criteria, resistance is indicated by mortality rates of less than 80% at 24 hrs post exposure, while mortality rates greater than 97% are indicative of susceptibility. Mortality rates between 80-97% indicate that resistance is suspected [17]. For probit analysis, knockdown times for 50% and 95% of the test population were estimated by the log time probit model using the Microsoft Excel Software 2007. The *kdr* allelic frequencies were calculated using the Hardy –Weinberg equation. Unpaired sample *t*-test was also used to determine the significant difference in mortality rates between each of the insecticides used using the SPSS software (version 15.0)

## Results

### Insecticide susceptibility

**Figure 2:**
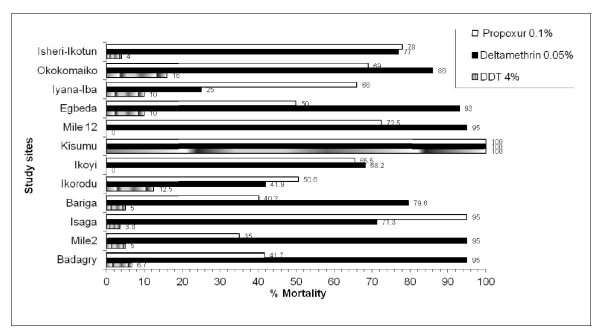
**Insecticide susceptibility status of*Anopheles gambiae s.l*in urban Lagos compared with the Kisumu strain.**

Overall, 2900 adult female mosquitoes were reared to adults from larval collections made in eleven localities. Of these, 2608 were morphologically identified as *A. gambiae s.l* and exposed to discriminating doses of DDT, deltamethrin and propoxur (Figure 1). All the field populations of *A. gambiae s.l* were resistant to DDT with mortality rates (≤ 16%) after a 24 hour recovery period (Figure 2). Resistance to deltamethrin was observed in *A. gambiae* populations in five localities; Isaga, Ikorodu, Ikoyi, Iyana Iba and Isheri – Ikotun. Mortality rates ranging between 25 -77% from propoxur exposure indicate resistance in ten localities excluding Isaga where resistance was suspected. Resistance to deltamethrin was also suspected in populations from Badagry, Mile 2, Bariga, Egbeda, Mile 12 and Okokomaiko, where mortality rates ranged between 80-95%. In summary, multiple resistance to three different classes of insecticides was observed in populations from five localities (Bariga, Ikorodu, Ikoyi, Iyana Iba and Isheri –Ikotun) (Figure 2).

Two populations of *A. gambiae* from Ikoyi and Mile 12 showed no mortality after one hour exposure to DDT. The overall results based on the WHO criteria for evaluating resistance suggests that the resistance status of *Anopheles gambiae* in urban Lagos vary with none of the populations exhibiting full susceptibility to any of the 3 insecticides tested.

### Knockdown effect of the insecticides

Percentage knockdown at 10 minute intervals was significantly higher in the susceptible Kisumu strain for all insecticides tested compared to all other samples from different localities (P < 0.05) in all cases. The log-time probit model used to estimate the KDT _50_ & KDT _95_ values (percentage knockdown with time) for DDT in Badagry, Mile 2, Isaga, Egbeda, Iyana Iba, Okokomaiko and Isheri-Ikotun study localities (Figure 1) indicated values that were unrealistic being greater than the mandatory 60 minutes recommended by the World Health Organization. All other KDT _50_ and KDT _95_ values of *A. gambiae* populations exposed to deltamethrin and propoxur within an hour were higher than those for the Kisumu susceptible by a factor of about 3–4 fold. Knock down time values higher than these are indicative of a very high level of resistance.

### Mortality against bendiocarb and propoxur in populations pre-exposed to piperonyl butoxide (PBO) synergist

**Figure 3:**
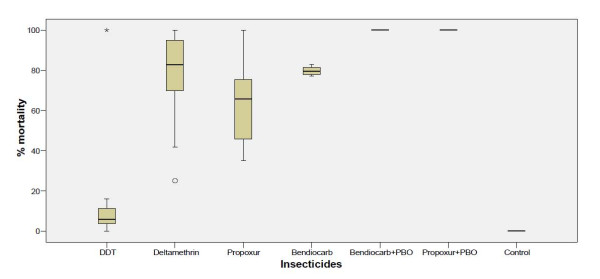
**Twenty- four hour post exposure mortalities of synergised and unsynergised*A. gambiae*exposed to different insecticides.**

The mortality rate observed in *A. gambiae* populations exposed to propoxur was lower (≤70%) compared with mortality rates (≤80%) in populations exposed to bendiocarb. However, a full susceptibility was observed in exposures to both insecticides when the *A. gambiae* populations were pre-exposed to PBO synergist (Figure 3).

### Species identification, molecular forms and frequencies of *kdr* and *ace -1*^*R*^ mutations

All mosquito samples (susceptible and resistant) drawn at random and analysed by species – specific PCR assays were identified as predominantly *A. gambiae s.s* and all belonged to the molecular M form (Figure 2). The knockdown resistance gene (*kdr*) was detected in all the *Anopheles* populations at various allelic frequencies ranging between 45%-77% (Figure 3). The genotypic frequencies showed that 134 (61%) and 79 (35.9%) mosquitoes were homozygous for the susceptible and resistance alleles. Only 4 (1.8%) carried the heterozygous alleles. The *kdr(e)* and ace-1^R^ mutation was absent in all the populations.

**Table 2:** **Species composition and frequency of molecular forms of*A. gambiae*mosquitoes exposed to different insecticides**

	**Insecticides**	**N**	***A.gambiae s.s***	**M forms**	**S forms**
	**DDT**	110	97	96	0
**Resistant samples**	**Deltamethrin**	95	91	91	0
	**Propoxur**	103	98	94	0
		308 (54.1%)			
	**DDT**	54	52	49	0
**Susceptible samples**	**Deltamethrin**	98	93	87	0
	**Propoxur**	110	97	93	0
		262 (45.9%)			
		570	528 (92.6%)*	510	0

*Eight percent of the samples failed to amplify.

N = Number of samples analysed.

**Table 3:** **The allelic frequencies of*kdr*mutation in molecular M forms of*A.gambiae s.s*in the study localities**

**Study localities**	*N	***kdr*mutation genotypes**			**Allelic frequency F(R)**
		**SS**	**RS**	**RR**	
**Badagry**	20	14	0	6	0.55
**Mile 2**	20	14	0	6	0.55
**Isaga**	20	8	0	12	0.77
**Bariga**	20	10	0	8	0.63
**Ikorodu**	20	9	0	11	0.74
**Ikoyi**	20	11	0	8	0.63
**Mile 12**	20	14	0	6	0.55
**Egbeda**	20	13	0	7	0.59
**Iyana-Iba**	20	14	2	4	0.45
**Okokomaiko**	20	14	0	6	0.55
**Isheri-Ikotun**	20	14	2	4	0.45
**Total**	**220**	**134 (60.9%)**	**4(1.8%)**	**79(35.9%)**	

*N = Number of *A. gambiae s.s* ‘M’ form analysed.

## Discussion

This study demonstrates the occurrence of resistance to deltamethrin, DDT and propoxur insecticides in populations of *A. gambiae* in urban Lagos. Previous studies have reported widespread resistance to DDT and pyrethroids in *Anopheles gambiae* from sub – Saharan Africa [24,25]. The existence of DDT resistance in all the urban communities surveyed is a confirmation of the spatio-temporal spread of DDT resistance in the urban communities of Lagos.

The co - occurrence of both DDT and deltamethrin resistance in the same *A. gambiae* population suggest a similar mechanism modulating for both pyrethroid and DDT resistance since both insecticides act on the same target site. This was confirmed with the identification of the *kdr* resistance mechanisms in all the communities. The discovery of carbamate resistance in 10 out of the 11 communities confirmed mosquito populations harbouring resistance to 3 classes of insecticides. Similar reports from studies carried out using field strains of *A. gambiae* populations in Guinea Conakry [26] and Congo [27] have demonstrated the occurrence of resistance to more than one class of insecticide. However, this is a first report of carbamate resistance in *A. gambiae s. s* in Nigeria, and evidence of its occurrence in populations already harbouring resistance to pyrethroids and organochlorines.

The existence of resistance to three classes of insecticides in populations of *A. gambiae* in urban Lagos is worrisome and an indication of the threatened sustainability of malaria vector control programmes utilising any of these insecticides. The major challenge arising from these results is that the choice of carbamate may not be a suitable alternative in the event of switching to another class of insecticide.

The identification of *A. gambiae s.s* and the molecular M forms in all samples analysed in Lagos is a clear indication of the predominance of these species in the urban communities where this study was carried out. These findings agree with earlier studies, which reported predominantly pure collections of *A. gambiae s.s* in south-western, Nigeria [13,28]. Interestingly, the species composition, the prevailing molecular M forms and the widespread incidence of the *kdr* resistance mechanism in the *A. gambiae* populations in Lagos share a strong similarity with *A. gambiae* populations from Ladji; an urban community in Benin Republic [29]. It is possible that there is a strong dispersal of mosquito species between Lagos and Benin republic owing to their location on the same transport corridor.

The higher disparity in mortalities observed in deltamethrin exposure compared with DDT suggest that the *kdr* alone may not be responsible for the resistance observed in these populations. Hence, the involvement of an additional operative resistance mechanism is possible. The leucine-serine point mutation (*kdr-e*), high levels of glutathione s-transferase and oxidase activities have been reported to confer higher resistance to DDT over pyrethroids [22,30]. In this study, the *kdr-e* mutation was found to be absent in the *A. gambiae* populations analysed. However, the synergistic assay confirmed the activity of metabolic enzymes, though not characterised, but may have increased resistance to DDT. Equally, the operative resistance mechanism responsible for the insensitivity of wild *A. gambiae s.s* to propoxur and bendiocarb was not as a result of mutation in the acetylcholinesterase target site reported from studies conducted in Burkina Faso and Cote d’Ivoire [31,32]. This was confirmed with the full restoration of susceptibility to carbamate in *A. gambiae* sub-populations pre-exposed to PBO synergists. Similar occurrence of carbamate resistance not associated with the G119S mutation has been reported in *A. gambiae s.s* from Congo [27], *Anopheles arabiensis from* Ethiopia [33] and in *A. funestus* from Mozambique [34].

Factors such as agricultural use of carbamate cannot be linked to selection for carbamate resistance as previously mentioned by other authors [29,32]. This is solely because the localities where these studies were conducted were non-agricultural areas. However, there is convincing evidence of the use of organophosphate based “locally made insecticides” by householders for personal protection in many of these localities (*unpublished results*). Hence, the carbamate resistance recorded may possibly have resulted from the elevated levels of esterases, which have been reported to be a primary mechanism involved in organophosphate and carbamate resistance [35,36].

## Conclusions

Carbamate may not be an alternative insecticide to substitute for the pyrethroid/DDT resistance occasioned in *A. gambiae* populations in Lagos. Hence, there is a need to incorporate resistance and integrated vector management approaches to malaria vector control in urban communities of Lagos, Nigeria.

## Competing interests

The authors declare that they have no competing interests.

## Authors’ contributions

OAO and TSA conceived and designed the study, AOO and ETI implemented the field study and carried out the susceptibility tests, MKO and AAA carried out the laboratory studies. MKO and JBO collated and analysed the data. AOO drafted the manuscript while TSA and OAO helped in editing the overall manuscript. All authors read and approved the final version of the manuscript.

## References

[B1] Gillies MT Coetzee M A supplement to the Anophelinae Africa South of the Sahara (Afrotropical region), Johannesburg, South Africa S Afr Inst Med Res 1987 55 1 143

[B2] Awolola TS Oduola AO Obansa JB Chukwura NJ Unyimadu JP Anopheles gambiae s.s breeding in polluted water bodies in urban Lagos, southwestern Nigeria J Vector Borne Dis 2007 44 241 244 18092529

[B3] Beier JC Keating J Githure JI Macdonald MB Impoinvil DE Novak RJ Integrated vector management for malaria control Malar J 2008 7 supp 1 10.1186/1475-2875-7-S1-S4 PMC2604879 19091038

[B4] Atieli HE Zhou G Afrane Y Lee M Mwanzo I Githeko AK Yan G Insecticide-treated net (ITN) ownership, usage, and malaria transmission in the highlands of western Kenya Parasit Vectors 2011 4 113 10.1186/1756-3305-4-113 21682919 PMC3135563

[B5] ScienceDaily. Retrieved July 19, 2011 http://www.sciencedaily.com/releases/2008/11/081118094632.htm

[B6] Devine GJ Ogusuku E Adaptability is key when monitoring insecticide resistance Bull World Health Organ 2009 87 887 887 10.2471/BLT.09.073502 20454474 PMC2789376

[B7] Zaim M Aitio A Nakashima N Safety of pyrethroid-treated mosquito nets Med Vet Entomology 2000 14 1 5 10.1046/j.1365-2915.2000.00211.x 10759305

[B8] Walker K Cost comparison of DDT and alternative insecticides for malaria control Med Vet Entomology 2000 14 345 354 10.1046/j.1365-2915.2000.00262.x 11129697

[B9] WHO Indoor Residual Spraying: Use of Indoor Residual Spraying for Scaling Up Global Malaria Control and Elimination: WHO Position Statement 2006 Global Malaria Programme, World Health Organization, Geneva

[B10] Eskenazi B Chevrier J Rosas LG Anderson HA Bornman MS Bouwman H Chen A Cohn BA de Jager C Henshel DS Leipzig F Leipzig JS Lorenz EC Snedeker SM Stapleton D The Pine River statement: human health consequences of DDT use Env Health Persp 2009 117 9 1359 1367 10.1289/ehp.11748 PMC2737010 19750098

[B11] Brogdon WG Mc Allister JC Insecticide Resistance and Vector Control Emerging Infect Dis 1998 4 4 606 613 10.3201/eid0404.980410 PMC2640263 9866736

[B12] Kristan M Fleischman H Della Torre A Stich A Curtis CF Pyrethroid resistance/Susceptibility and differential urban/rural distribution of Anopheles arabiensis and A. gambiae s.s malaria vectors in Nigeria and Ghana Med Vet Entomol 2003 17 326 332 10.1046/j.1365-2915.2003.00449.x 12941018

[B13] Awolola TS Brooke BD Hunt RH Coetzee M Resistance of the malaria vector Anopheles gambiae s.s to pyrethroid insecticide in south western- Nigeria Ann Trop Med and Parasitology 2002 96 849 852 10.1179/000349802125002581 12625942

[B14] Awolola T Oduola A Oyewole IO Obansa JB Amajoh C Koekemoer L Coetzee M Dynamics of knockdown pyrethroid insecticide resistance alleles in a field population of Anopheles gambiae s.s. in southwestern Nigeria J Vect Borne Dis 2007 44 181 188 17896620

[B15] Oduola AO Obansa JB Ashiegbu CO Adeogun A Otubanjo OA Awolola TS High level of DDT resistance in the malaria mosquito: Anopheles gambiae s.l. from rural, semi urban and urban communities in Nigeria J Rural Trop Pub Health 2010 9 114 120

[B16] Gillies MT Coetzee M A Supplement to the Anophelinae of Africa South of the Sahara In Publication of South African Institute of Med Res 1987 55 1 143

[B17] World Health Organisation Test procedures for Insecticide Resistance in Malaria Vectors, Bio-efficacy and Persistence of Insecticides on Treated Surfaces. (WHO/CDS/CPC/MAL/98.12) 1998 WHO Geneva, Switzerland

[B18] Abott WS A method of computing the effectiveness of an insecticide J Am Mosq Control Assoc 1987 3 302 303 3333059

[B19] Collins FH Mendez MA Rasmussen MO Mehaffey PC Besansky NJ A ribosomal RNA gene probe differentiates member species of the Anopheles gambiae complex AmJTrop Med Hyg 1987 37 37 41 10.4269/ajtmh.1987.37.37 2886070

[B20] Fanello C Della Torre A Santolamazza F Simultaneous identification of species and molecular forms of the Anopheles gambiae complex by PCR-RFLP Med Vet Entomol 2002 16 461 464 10.1046/j.1365-2915.2002.00393.x 12510902

[B21] Martinez-Torres D Chandre F Williamson MS Darriet F Berge JB Devonshire AL Guillet P Pasteur N Pauron D Molecular characterization of pyrethroid knockdown resistance (kdr) in the major malaria vector Anopheles gambiae s.s Insect Mol Biol 1998 7 179 184 10.1046/j.1365-2583.1998.72062.x 9535162

[B22] Ranson H Jensen B Vulule JM Wang X Hemingway J Collins FH Identification of a point mutation in the voltage-gated sodium channel gene of Kenyan Anopheles gambiae s.s. associated with resistance to DDT and pyrethroids Ins Mol Biol 2000 9 491 497 10.1046/j.1365-2583.2000.00209.x 11029667

[B23] Weill M Malcolm C Chandre F Mogensen K Berthomieu A Marquine M Raymond M The unique mutation in Ace-1 giving high insecticide resistance is easily detectable in mosquito vectors Insect Mol Biol 2004 13 1 7 10.1111/j.1365-2583.2004.00452.x 14728661

[B24] Himeidan YE Abdel Hamid MM Jones CM Ranson H Extensive permethrin and DDT resistance in Anopheles arabiensis from eastern and central Sudan Parasit Vectors 2011 4 154 10.1186/1756-3305-4-154 21812972 PMC3170279

[B25] Rogan WJ Chen A Health risks and benefits of bis(4-chlorophenyl)-1,1,1-trichloroethane (DDT) Lancet 2005 366 9487 763 773 10.1016/S0140-6736(05)67182-6 16125595

[B26] Vezenegho SB Brooke BD Hunt RH Coetzee M Koekemoer LL Malaria vector mosquito composition and insecticide susceptibility status in Guinea Conakry, West Africa Med Vet Entomol 2009 23 326 334 10.1111/j.1365-2915.2009.00840.x 19941598

[B27] Koekemoer LL Spillings BL Christian RN Lo TM Kaiser ML Norton RAI Oliver SV Choi KS Brooke BD Hunt RH Coetzee M Multiple Insecticide Resistance inAnopheles gambiae(Diptera: Culicidae) from Pointe Noire, Republic of the Congo. Vect. Borne Zoo Dis 2011 "10.1089/vbz.2010.0192" 10.1089/vbz.2010.0192 21417925

[B28] Onyabe DY Vajime CG Nock IH Ndams IS Akpa AU Alaribe AA Conn JE The distribution of M and S molecular forms of Anopheles gambiae in Nigeria Trans R Soc Trop Med Hyg 2003 97 605 608 10.1016/S0035-9203(03)80045-7 15307438

[B29] Yadouleton AW Padonou G Asidi A Moiroux N Bio-Banganna S Corbel Corbel Gbenou D Yacoubou I Gazard K Akogbeto MC Insecticide resistance status in Anopheles gambiae in southern Benin Malaria J 2010 9 83 10.1186/1475-2875-9-83 PMC2858214 20334637

[B30] Ranson H Jensen B Wang X Prapanthadara L Hemingway J Collins FH Genetic mapping of two loci affecting DDT resistance in the malaria vector Anopheles gambiae Ins Mol Biol 2000 9 499 507 10.1046/j.1365-2583.2000.00214.x 11029668

[B31] Ahoua Alou LP Koffi AA Adja MA Tia E Kouassi PK Koné M Chandre F Distribution of ace-1R and resistance to carbamates and organophosphates in Anopheles gambiae s.s populations from Côte d'Ivoire Malaria J 2010 9 167 10.1186/1475-2875-9-167 PMC2908637 20553593

[B32] Dabire KR Diabate A Djougbenou A Ouari A N’Guessan R Ouédraogo J Hougard J Chandre F Baldet T Dynamics of multiple insecticide resistance in the malaria vector Anopheles gambiae in a rice growing area in South- Western Burkina Faso Malaria J 2008 7 188 10.1186/1475-2875-7-188 PMC2564969 18817564

[B33] Yewhalaw D Wassie F Steurbaut W Spanoghe P Van Bortel W Multiple Insecticide Resistance: An Impediment to Insecticide-based Malaria Vector Control Programme PLoS One 2011 6 1 e16066 10.1371/journal.pone.0016066 21264325 PMC3020220

[B34] Cuamba N Morgan JC Irving H Steven A Wondji CS High Level of Pyrethroid Resistance in an Anopheles funestus Population of the Chokwe District in Mozambique PLoS One 2011 5 6 e11010 20544036 10.1371/journal.pone.0011010 PMC2882342

[B35] Hemingway J Karunaratne SHPP Mosquito carboxylesterases: a review of the molecular biology and biochemistry of a major insecticide resistance mechanism Med. Vet. Entomol 1998 12 1 12 10.1046/j.1365-2915.1998.00082.x 9513933

[B36] Hemingway J Hawkes NJ McCarroll L Ranson H The molecular basis of insecticide resistance in mosquitoes Insect Biochem Mol Biol 2004 34 7 653 665 10.1016/j.ibmb.2004.03.018 15242706

